# Pea in the Trachea

**Published:** 1907-07

**Authors:** 


### Pea in the Trachea.

Dr. W. P. Nicholson reported the history of a child 18 months old with a "whip-poor-will" pea in its trachea. There were paroxysmal attacks of coughing but no abnormal sounds could be detected by carefully listening with a stethoscope. On this account he first hesitated as to the advisability of operating, but finally decided to do it. The patient came very near dying several times during the operation, but persistent artificial respiration and careful wiping away of the pus that was occasionally coughed up, kept the child alive until the pea was finally recovered. The trachea was kept open for several days and there was a considerable flow of pus from it.

A point to be remembered is that one cannot be guided alone by the sounds in locating a foreign body, as this gave none.

### COMPOUND FRACTURE OF THE SKULL.

Dr. W. S. Goldsmith reported a compound fracture of the skull in which he had used one of Dr. Nicholson's celluloid plates to fill in the space left after removing the bone. The patient also had a fractured spine. The skull wound is doing well, but it was necessary to leave a small drain in it and Dr. Goldsmith asked Dr. Nicholson as to the effect of such drainage and whether or not it would make the prognosis uncertain.

Dr. Nicholson replied that he had not used these plates in a compound fracture of the skull, but did not see why it should not be most satisfactory. He always leaves a small opening for the escape of fluid from the cavity. One patient with infection around the plate had given him a great deal of trouble and several plastic operations had been necessary to cover the plate completely. It was finally removed and instead of finding the brain raw, ragged and inflamed as it was when the first operation had been done to remedy the defect, it was covered by a firm, smooth coating very much like dura, and believes nothing else could have done so well. He thinks the operation Dr. Goldsmith did will do more than anything else could do to prevent hernia of the brain immediately and adhesions, pressure and epilepsy ultimately.

---

The new state cancer laboratory, Buffalo, has been granted another appropriation of $20,000 by the legislature, for the continued investigation into the cause of cancer.

---

In the treatment of the chronic skin inflammations, following in the wake of attacks of toxic dermatitis, attention to the general condition of the health, avoidance of anything irritating to the skin, a carefully selected diet and proper care of the skin are important features which must not be neglected. In addition, Battle's preparation of echinacea augustifolia and thuja occidentalis, which goes under the trade name of Ecthol, should be used both locally and internally, a drachm should be taken four times a day. —American Journal Dermatology.

